# Can Environmental Regulation Reduce Labor Costs and Improve Business Performance? Evidence From the Air Quality Index

**DOI:** 10.3389/fpubh.2019.00398

**Published:** 2020-01-17

**Authors:** Kuang-Cheng Chai, Yang Huang, Ke-Chiun Chang, Wen-Jun Hu

**Affiliations:** ^1^Business School, Guilin University of Electronic Technology, Guilin, China; ^2^School of Economics and Management, Wuhan University, Wuhan, China

**Keywords:** environmental regulation, air quality, employee compensation, labor costs, corporate performance

## Abstract

This study examines selected companies in China from 2012 to 2017 matched with provincial air quality index data and uses ordinary least squares regression to examine the relationship between environmental regulation, air quality, employee compensation, and corporate performance. The study found that, first, environmental regulation has eliminated heavy polluting enterprises and promoted the upgrading of enterprise equipment through the cost increase effect, thereby improving regional air quality. Second, good air quality can increase non-monetary benefits for employees, so that corporate employees' monetary compensation can be kept at a low level. Third, in the aforementioned context, with the significant reduction in employee compensation, corporate performance has increased. This study expands the research on the impact of environmental regulation on corporate behavior and enriches the theoretical literature on employee compensation management. Furthermore, to alleviate the adverse effects of high employee compensation on corporate performance under severe air quality, it provides recommendations moving forward. In addition, this study provides empirical evidence for the development of the concept of “lucid waters and lush mountains are invaluable assets” from the perspective of labor cost.

## Introduction

Since the economic reform and opening up in 1978, China's economy has made significant progress. However, extensive reliance on production input has not been effectively transformed, causing considerable damage to China's ecological environment. With increasing pollution prevention and control work, environmental protection and economic growth have become a critical dilemma facing China's economic and social development. Under the increasingly severe situation of resource constraints in China, the extensive growth model with high-energy consumption and high pollution is gradually declining. Green development is the source of economic growth and an important way to cultivate competitive advantage and improve economic strength. As a microproduction subject, enterprises are important carriers for economic transformation and promoting green development (1–3). In this process, the relationship between environmental regulation and corporate performance is becoming more and more compelling. The implementation of environmental regulation, through what channels affect the performance of enterprises? How does it affect business performance? This is the next question worth considering.

Many scholars have actively explored the above topics. Jaffe et al. (4) found that environmental regulation would bring a “crowding out effect” to enterprises; that is, enterprises that would invest in other aspects of the investment after meeting the requirements of environmental regulation would eventually hinder the productivity of the company. While, Porter and Welsh Brown (5) acknowledges the existence of direct costs, he believes that enterprises will promote technological innovation in the face of environmental regulation, resulting in “innovation advantage” and “first mover advantage,” thus achieving a win–win situation for both the environment and economy. Dean and Brown (6) verified that environmental regulation would increase industry barriers to new entry into the market. As the production cost of enterprises increases with investment in machinery and equipment, the threshold for new enterprises to enter the industry will increase. Thus, companies that have been established earlier and have previously entered the industry benefit from the “industry barrier effect” and therefore benefit from it.

Buysse and Verbeke (7) and Cabugueira Manuel (8) argue that the impact of environmental regulation on firm performance depends on the management's perception of the importance of environmental regulation. This is consistent with the conclusions of Stone et al. (9): the more the top management pays attention to environmental issues, the greater the company's investment in environmental issues. They believe that environmental regulation is a company's opportunity; they may adopt new technologies, strengthening the cooperation of stakeholders and other means to create value for the enterprise. In contrast, Sharma and Vredenburg (10) show that if the management believes environmental regulation is a corporate threat, they will resist, limiting innovation and change. Pashigian (11) argues that the size of the firm also affects the role of environmental regulation in corporate performance. Because, faced with the financial burden brought by the same intensity of environmental regulation, the ability of small enterprises to bear the burden is far less than that of large enterprises. Consequently, it is more difficult for small enterprises to compete with large enterprises, and it is more difficult to create value. Bai et al. (12) and Duanmu et al. (13) believe that high market integration and market competition are conducive to the development of technology intermediary market, which is conducive to the role of energy-saving and emission reduction in technological innovation while improving energy efficiency. Increased utilization may drive business performance.

Previous research on environmental regulation and corporate performance mainly discusses the impact of environmental regulation on corporate investment, technological innovation, industrial barriers, and other factors such as environmental regulation and management perception, corporate size, and age. Therefore, this study combines employee compensation management from the perspective of non-monetary benefits to explore the impact of environmental regulation on corporate performance, enriching the existing research. This study specifically explores the relationship between environmental regulation, air quality, employee compensation, and corporate performance as the previous literature rarely considers the environmental benefits brought by environmental regulation and the employee compensation management behavior in the context of environmental benefits. Moreover, existing research on compensation focuses on executive compensation, and research on ordinary employee compensation is rare. In fact, although the average salary level of each employee is relatively low, the total employee compensation and total labor cost of the company account for a large proportion of the company's income, and its changes will have a greater impact on the company's production and operation, which deserves people's attention.

Chen et al. (14) have shown that, at a given shadow economy level, stricter environmental controls will help reduce pollution. In addition, an increase in the proportion of corrupt officials may undermine the environmental governance of environmental regulations. Improvement of air quality will help improve the physical and mental health of employees and improve their work efficiency and quality of life. Skrzypek et al. (15) believes that, with the reduction in health costs, employees' enthusiasm for participating in work will be greatly enhanced. Therefore, good air quality is an important non-monetary benefit for employees. Jensen and Murphy (16) support these findings. Such non-monetary benefits to employees in areas with severe air pollution are low; Cole et al. (17) believe that low non-monetary gains can lead to strong willingness to leave high-skilled employees in heavily polluted areas. In such a scenario, to retain employees, enterprises will increase their monetary compensation to compensate for the low non-monetary benefits caused by poor air quality. This will significantly increase the cost of business and adversely affect the company's performance. On the contrary, Myers (18) shows that good air quality as a non-monetary incentive offsets the motivation of employees to ask for salary increases, which is conducive to the reduction in corporate costs and improves corporate performance.

Based on this, this study explores whether environmental regulation can affect the relationship between air quality and employee compensation and whether the resulting changes in employee compensation (labor cost) can have a significant impact on business performance. This study is based on the following three aspects. First, poor air quality will affect the employees' physical and mental health, so they cannot enjoy the non-monetary benefits brought by good air. Second, due to the substitution relationship between non-monetary income and monetary compensation, when regional environmental quality is improved, the non-monetary benefits of employees can be improved, which may have an impact on the employees' monetary compensation. Third, environmental regulation is an effective measure to improve air quality. Therefore, while environmental regulations improve the non-monetary benefits of employees, they also reduce the compensation for corporate monetary compensation, which will help enterprises reduce costs, which in turn may help companies improve their performance.

The contributions of this study are as follows. First, the existing literature on environmental regulation mainly focuses on the impact of relevant measures on the technological innovation and investment behavior of enterprises. This study takes the employee compensation as the starting point and empirically analyzes the effect of environmental improvement brought by environmental regulation on the non-monetary benefits of employees, which will help identify and evaluate the impact of environmental regulation on enterprise development. Second, the conclusions of this study also contribute to a deeper understanding of the government's environmental regulation and support the Chinese government's concept of “lucid waters and lush mountains are invaluable assets” repeatedly proposed in recent years to understand and optimize environmental protection. Finally, the relationship between economic development and the in-depth implementation of environmental governance has certain reference value.

## Literature Review and Hypotheses

### Environmental Regulation and Employee Compensation

Poor air quality can negatively affect the physical and mental health of employees (19–21), increasing the cost of employee work. If the employee's income remains the same, as the cost increases, their mental state will change, which may result in increased absenteeism (19). To motivate employees to work hard, companies are forced to internalize the external costs caused by air pollution, leading to increased employee compensation. Good air quality can effectively reduce external costs caused by air pollution and help companies reduce production costs and reduce employee compensation.

Environmental regulation is an effective mechanism to improve air quality. Through local environmental regulations, emissions of harmful substances such as exhaust gas will be reduced, thereby improving air quality. As air quality improves, it will reduce the external costs caused by pollution and increase the employees' non-monetary benefits. Moreover, there is a mutual substitution and a trade-off between non-monetary returns and monetary compensation. Once air quality is improved, the monetary compensation given to employees may be reduced. In summary, this study proposes research hypothesis 1.

H1: Under other conditions, areas with strong environmental regulations have low employee compensation levels.

### Environmental Regulation and Air Quality

If environmental regulations are implemented in an area, it will first affect the cost increase in the enterprise, which in turn will affect the environmental quality of the region. Once the relevant environmental regulation measures are issued, the most direct impact is an increase in the relative prices of the production factors of polluting enterprises through taxes, regulations, and other policy measures, and an increase in the production costs associated with enterprises (22–24). If the company cannot reduce the emission of harmful gases, its production costs will remain high for a long time. Polluting companies will be forced to move out of the region when their income cannot be significantly increased to cope with the rising costs. The local regional air quality is thus improved. Second, environmental regulations have strengthened the power of enterprises to reform and innovate environmental protection and energy-saving technologies. To reduce the corresponding production costs, those enterprises that can stay will inevitably carry out environmental protection transformation and technological innovation for relevant production equipment, carry out clean production, and reduce harmful gas emissions (5), thus achieving improvement in local areas. Regardless of the mechanism of action, environmental regulations will reduce local environmental pollution and improve environmental quality, such as air quality. The results of Acemoglu et al. (25) show that the implementation of pollution prevention policies, including “carbon tax” and technical subsidies, can bring about the upgrading and sustainable growth of related technologies. Based on this, this paper proposes research hypothesis 2.

H2: The air quality is better in areas where environmental regulations are stronger, under other conditions.

### Air Quality and Employee Compensation

According to Maslow's hierarchy of needs (26), after the most basic physiological needs are met, workers will seek high-level security needs, such as good health. Poor air quality increases their risk of illness (27, 28). Ordinary employees usually require companies to pay the corresponding “environmental injury compensation” to make up for unmet health and safety needs. In addition, according to the “economic man” hypothesis, trade unions weigh the pros and cons between air quality and monetary compensation, and there is a trade-off between the two. In the case of other external environments, if the air quality of a certain place becomes increasingly worse, the health and safety requirements of employees are increasingly difficult to guarantee, which means that the non-monetary benefits of employees become fewer. At this point, employees will demand increased monetary compensation from the company as a corresponding compensation. Panasonic's “pollution allowance” and Coca-Cola's “haze danger subsidy” are some examples. Conversely, the better the air quality, the higher the non-monetary benefits of the employees and the lower the monetary compensation. Based on this, this paper proposes a third research hypothesis.

H3: Under other conditions, the better the air quality of the listed company's location, the lower the employee's salary level.

### Employee Compensation and Corporate Performance

Anderson (29) believes that employee compensation is closely linked to corporate performance. Employee compensation affects corporate performance in two ways (30): cost burden and employee incentives. Specifically, employee compensation is not only a material cost to the use of human resources but also an incentive for employees. Reasonable employee compensation enables the company to complete incentives for employees when it bears a certain amount of labor costs. When the employee's salary exceeds a reasonable level, it means that the material salary paid by the enterprise is greater than the incentive effect generated by the company. As a result, the labor cost of the enterprise is too heavy to make ends meet, which in turn has an adverse impact on the enterprise.

Environmental regulation measures improve the air quality of the region and increase the non-monetary benefits of employees. These non-monetary benefits can also have incentives for employees. Moreover, these companies do not need to use monetary compensation to compensate for losses on non-monetary returns, as in companies with poor air quality. This allows companies in areas with good air quality to achieve the same level of incentives while reducing labor costs. Therefore, low employee compensation for companies with good air quality can improve corporate performance. As a result, this paper proposes the fourth research hypothesis.

H4: For enterprises that need to make up for the loss of non-monetary income, the low employee compensation of enterprises with good air quality is conducive to the improvement of corporate performance under other conditions.

**Figure 1:**
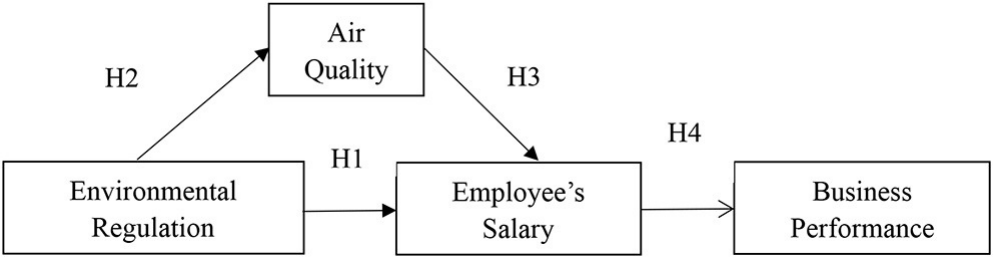
Research framework.

According to this theoretical analysis and research hypothesis, the analytical framework is constructed as shown in Figure 1.

## Research Design

### Data Source and Processing

This study selects A-share listed companies from 2012 to 2017 as samples. The data on environmental regulation and air quality are taken from the relevant statistical yearbooks where the enterprises are registered. The specific data sources are the China Urban Competitiveness Yearbook and the China Environmental Statistics Yearbook. The microfinancial data of the enterprises used in this paper are all from the CSMAR database. The data of other variables are from the China Energy Statistical Yearbook, the China Statistical Yearbook, and the China Construction Industry Statistical Yearbook. The samples were screened using the following conditions: (1) excluding missing samples of the relevant variables; (2) excluding the special treatment (ST, which means the company's operation losses for two consecutive years; risk warning) and particular transfer (PT, which means the company's operation losses for three consecutive years; delisting warning) samples and finally obtaining 21,372 samples. To eliminate the interference of outliers, winsorize tailing processing is performed on the 1 and 99% quantiles of continuous variables.

### Variable Definition and Measurement Model

#### Variable Definition

The definitions of related variables are presented in Table 1. Model core variables include employee compensation, air quality, business performance, and environmental regulation. Employee compensation (Lgwage) refers to Core et al. (31) and Hayes and Schaefer (32) on the calculation method of employee compensation; this article uses the cash flow statement “payment to employees and cash paid for employees” minus total executive compensation, except taking the number of employees on the job, and taking the natural logarithm as a substitute for ordinary employees' salaries. Air quality (Air) is derived from the three-level indicator of the China Urban Competitiveness Yearbook Environmental Quality Index. The index is distributed between 0 and 1. The larger the index, the better the air quality in the region. The enterprise performance is measured in two dimensions, one is the financial performance indicator (ROE), and the net profit is divided by the average shareholders' equity. The other dimension is the growth indicator, which is measured by the growth rate of the company's operating income. There are many measures for environmental regulation (hr). The emission intensity of different pollutants used by Domazlicky and Weber (33) represents the intensity of environmental regulation. The gross domestic product (GDP)/enterprise energy consumption adopted by Jiang and Lu (34) and Jiang et al. (35) is based on linear pollution standardization of unit pollution emissions and weighted average consolidation. This paper considers the error caused by avoiding the heterogeneity between industries and draws on the practices of Song and Wang (36), from the perspective of pollution control costs, the selection of local industrial pollution control investment, and local production. This is the ratio to measure environmental regulation indicators.

**Table 1:** Definitions of variables.

**Variables**	**Symbol**	**Variable metrics**
Employee's salary	Lgwage	Deducting the number of executives/employees in cash paid to employees and for employees (this value is taken by natural logarithm)
Air quality	Air	The environmental quality index is between 0 and 1. The larger the air quality, the better
Business performance	Roe	Net profit/total assets
Environmental regulation	Hr	Local industrial pollution control investment/local GDP
Company size	Size	Natural logarithm of total assets at the end of the period
Assets and liabilities	Lev	Total liabilities at the end of the period divided by total assets at the end of the period
Cash holding level	Cash	Initial monetary funds after standardization of total assets
Executive compensation	Lgcom	Natural logarithm of executive compensation
Equity concentration	Shr	The shareholding ratio of the largest shareholder
Nature of property	Soe	State-owned enterprise is 1, otherwise 0
Industrial output value	InGDP	Local industrial GDP accounts for the proportion of total local GDP
Coal consumption	CCons	Total coal consumption by region
Motor vehicle possession	Vepop	Total amount of diesel locomotives (including motorcycles, cars, trucks)
Construction area	Buiope	Building area of each district

Model control variables refer to previous research on the factors affecting employee compensation and firm performance (37–40). This paper controls the following company characteristic variables to alleviate the endogenous problem caused by missing variables: company size (Size), using natural logarithm measurement of total assets at the end of the period; asset/liability ratio (Lev), which is the total used-to-end liabilities divided by total assets at the end of the period; cash holding level (Cash), using total assets standardization after the beginning of the period of monetary capital measurement; executive compensation (Lgcom), using the logarithm of total executive compensation; equity concentration (Shr), using the largest shareholder share ratio as a proxy variable; and state-owned enterprise dummy variable (Soe). If it is a state-owned enterprise, the variable is 1, otherwise 0. In addition, to control the possible factors affecting air quality, this study refers to the relevant literature (41–43) and controls the following variables in the model. The industrial GDP of the city where the enterprise is located accounts for the proportion of total GDP (InGDP), dividing the local industrial GDP by the proportion of local GDP; coal consumption (CCons), total coal consumption in various places; vehicle ownership (Vepop), diesel locomotives in various places (including motorcycles, automobiles), the total amount of trucks and the construction area (Buiope), and the total building area of each district.

#### Regression Model

To test hypothesis H1, this paper takes employee compensation and environmental regulation as the explanatory variables and controls the relevant enterprise characteristics influencing factors to establish a multiple regression model (1) to verify H1:

(1) $$\begin{array}{l} \text{Lgwage}\;=\alpha_{0}+\alpha_{1}\text{Hr}+\alpha_{2}\text{Size}+\alpha_{3}\text{Lev}+\alpha_{4}\text{Roe}\;\\ \text{ + }\alpha_{5}\text{Cash}+\alpha_{6}\text{Lgcom}+\alpha_{7}\text{Shr}+\alpha_{8}\text{Soe}\;\\ \text{ + }\alpha_{9}\text{Ccons}+\alpha_{10}\text{Vepop}+\alpha_{11}\text{Buiope}+\varepsilon \end{array}$$

In model (1) of this paper, the coefficient α_1_ measures the impact of environmental regulation on employee compensation levels. The focus of model (1) is the symbolic and saliency level of α_1_. As mentioned above, in theory, environmental regulations are an important means to improve air quality, which can reduce the uncertainty and risk faced by employees in the region. Those can increase the non-monetary benefits of the company, thereby reducing labor costs. If the inference in this paper is correct, then α_1_ will be significantly <0.

To test hypothesis H2, this study uses air quality and environmental regulation as the explanatory variables and controls the related macroscopic influencing factors to establish a multiple regression model (2) to verify H2:

(2) $$\begin{array}{l} \text{Air}=\beta_{0}+\beta_{1}\text{Hr}+\beta_{2}\text{InGDP}+\beta_{3}\text{CCons}+\beta_{4}\text{Vepop}\;\\ \text{ + }\beta_{5}\text{Buiope}+\varepsilon \end{array}$$

In model (2) of this paper, the coefficient β_1_ measures the effect of environmental regulation on air quality. The focus of model (1) is the symbolic and saliency level of β_1_. The implementation of relevant environmental regulation measures will bring about cost increase effect and environmental protection technology improvement, thus reducing regional air pollution. If the inference in this paper is correct, then β_1_ will be significantly >0.

To test hypothesis H3, this paper takes employee compensation as the explanatory variable, air quality index as explanatory variable, and controls the related factors of enterprise characteristics to establish multiple regression model (3) to verify H3:

(3) $$\begin{array}{l} \text{Lgwage}={ϒ}_{0}+{ϒ}_{1}\text{Air}+{ϒ}_{2}\text{Size}+{ϒ}_{3}\text{Lev}+{ϒ}_{4}\text{Roe}\\ \;+{ϒ}_{5}\text{Cash}+{ϒ}_{6}\text{Lgcom}+{ϒ}_{7}\text{Shr}\;\text{+}{ϒ}_{8}\text{Soe}\\ \;+{ϒ}_{9}\text{Ccons}+{ϒ}_{10}\text{Vepop}+{ϒ}_{11}\text{Buiope}+\varepsilon \end{array}$$

In model (3) of this paper, the coefficient ϒ_1_ measures the impact of air quality on employee compensation levels. The focus of model (3) is the symbolic and saliency level of ϒ_1_. The improvement of air quality will bring non-monetary benefits, and under the same conditions, non-monetary income and monetary compensation will be a fluctuant process the better the air quality, the lower the employee compensation level. If the inference in this paper is correct, then ϒ_1_ will be significantly <0.

To test hypothesis H4, this paper sets the enterprise performance and the environmental regulation as the explanatory variables and the related enterprise characteristic as influence variable. The established multiple regression model (4) verifies H4:

(4) $$\begin{array}{l} \text{Performances}\;\;=\;\;\delta_{0}+\delta_{1}\text{Lgwage}+\delta_{2}\text{Size}+\delta_{3}\text{Lev}\\ \;+\delta_{4}\text{Cash}+\delta_{5}\text{Lgcom}+\delta_{6}\text{Shr}+\delta_{7}\text{Soe}\;\\ \text{ + }\delta_{8}\text{Ccons}+\delta_{9}\text{Vepop}+\delta_{10}\text{Buiope}+\varepsilon \end{array}$$

In model (4) of this paper, the coefficient δ_1_ measures the impact of employee compensation levels on firm performance. The focus of model (4) is the sign and significance level of δ_1_. Good air quality brings non-monetary benefits and forms an alternative to monetary compensation. Therefore, under the premise of reducing labor cost expenditure, the same intensity of incentive effect can be obtained, which can improve corporate performance. Enterprise performance is measured by financial performance indicators and corporate growth indicators. If the inference in this paper is correct, then δ_1_ will be significantly >0.

This paper uses the ordinary least squares regression to estimate the above measurement model. Considering the robustness of the conclusions, robust standard errors were used in the estimation. At the same time, to alleviate endogeneity problems caused by missing variables, we used industry, provinces, and corporate fixed effects.

## Empirical Analysis

### Descriptive Statistics and Correlation Analysis

Table 2 presents the descriptive statistics of the main variables in this study. The minimum value of enterprise employee compensation (Lgwage) in the sample is 10.4525, and the maximum value is 13.1674, which indicates that the gap between high- and low-paid employees is large, and the average value is 11.5455. The number of digits is 11.4796, and the standard deviation is 0.5158. This indicates that, although there are differences in employee compensation levels, this difference is not obvious or common in sample companies. The extreme values of salary levels are relatively concentrated. The minimum air quality (Air) is 0.0700, and the maximum is 0.7020, indicating a large difference in air quality among regions; the mean is 0.2082, the median is 0.1500, and the standard deviation is 0.1381, which indicates the air quality in different regions. Large differences are common. The air quality index of most of the samples is mainly concentrated between 0.07 and 0.2580, indicating that the areas affected by air pollution in China are more extensive, and the situation of pollution prevention and control is grim. The minimum environmental regulation (Hr) is 0.0002, and the maximum value is 0.0111, indicating that the environmental regulation intensity varies greatly from region to region; its mean value is 0.0012, the median is 0.0008, and the standard deviation is 0.0016, which indicates environmental regulation differences are significant in different regions.

**Table 2:** Descriptive statistics.

**Variables**	***N***	**Mean**	**SD**	**Min**	**Max**
Lgwage	21,372	11.5455	0.5158	10.4525	13.1674
Air	21,372	0.2082	0.1381	0.0700	0.7020
Hr	21,372	0.0012	0.0016	0.0002	0.0111
Roe	21,327	0.0469	0.0675	−0.2505	0.2536
Size	21,327	21.9408	1.5168	19.0581	26.9609
Lev	21,372	0.4345	0.2128	0.0539	0.9401
Cash	21,327	0.1609	0.1222	0.0006	0.6069
Lgcom	21,372	15.2827	0.7456	13.4027	17.4254
Shr	21,372	0.3078	0.1839	0.0000	0.7738
Soe	21,372	0.2970	0.4570	0.0000	1
InGDP	21,372	0.4361	0.0585	0.2460	0.5460
CCons	21,372	102.1077	0.5288	100.9600	103.3000
Vepop	21,372	4659.7680	9958.4060	25	72,530
Buiope	21,372	18.5412	3.8030	12	32

Table 3 reflects the correlation between the variables. Among them, the largest correlation coefficient is that between the company size (Size) and the vehicle possession (Vepop), which is 0.599. This indicates that the correlations between the variables in the model do not lead to serious multicollinearity problems. That is, multicollinearity will not be a distracting factor in the empirical results of this paper.

**Table 3:** Pearson correlation coefficient.

**Variables**	**Lgwage**	**Air**	**Hr**	**ROE**	**Size**	**Lev**	**Cash**	**Lgcom**	**Shr**	**Soe**	**InGDP**	**CCons**	**Vepop**
Lgwage	1												
Air	0.056^***^	1											
Hr	−0.074^***^	0.096^***^	1										
ROE	−0.002	0.005	−0.071^***^	1									
Size	0.343^***^	0.052^***^	0.012^*^	−0.233^***^	1								
Lev	0.151^***^	−0.008	0.049^***^	−0.358^***^	0.478^***^	1							
Cash	0.059^***^	−0.031^***^	−0.047^***^	0.083^***^	−0.170^***^	−0.291^*^	1						
Lgcom	0.376^***^	−0.003	−0.109^***^	0.142^***^	0.551^***^	0.191^*^	−0.033^***^	1					
Shr	0.052^***^	0.037^***^	0.017^**^	−0.105^***^	0.318^***^	0.023^*^	0.023^***^	0.006	1				
Soe	0.198^***^	0.041^***^	0.067^***^	−0.169^***^	0.380^***^	0.243^*^	−0.037^***^	0.085^***^	0.270^***^	1			
InGDP	−0.270^***^	−0.092^***^	−0.053^***^	0.062^***^	−0.111^***^	0.005	−0.031^***^	−0.118^***^	−0.073^***^	−0.052^***^	1		
CCons	−0.051^***^	−0.305^***^	−0.001	0.050^***^	−0.098^***^	0.038^*^	0.077^***^	−0.039^***^	−0.068^***^	0.006	0.070^***^	1	
Vepop	0.073^***^	0.000	−0.033^***^	−0.061^***^	0.599^***^	0.274^*^	−0.082^***^	0.345^***^	0.184^***^	0.212^***^	−0.053^***^	−0.003	1
Buiope	0.142^***^	0.008	0.039^***^	−0.008	0.484^***^	0.283^*^	−0.097^***^	0.459^***^	0.033^***^	0.299^***^	0.037^***^	0.050^*^	0.355^***^

* *p < 0.01*,

** *p < 0.05*,

*** *p < 0.1*.

### Regression Analysis

It can be seen from Table 4 (1) that after controlling the relevant factors affecting employee compensation, environmental regulation, and employee compensation are significantly negatively correlated at the 1% level. In columns (2)–(4), the industry's, province's, and firm's fixed effects are further controlled, and environmental regulations and employee compensation are still significantly negatively correlated at the 1% level. This explains that in other areas where the conditions are the same, in areas with high environmental regulation intensity, the salary level of local enterprises will be lower than that of other places, and H1 is proven.

**Table 4:** Relationship between environmental regulation and employee compensation.

**Variables**	**Random effect**			**Fixed effect**
	**Lgwage (1)**	**Lgwage (2)**	**Lgwage (3)**	**Lgwage (4)**
Hr	−15.740^***^	−14.090^***^	−16.790^***^	−14.590^***^
	[3.3982]	[3.3365]	[4.1338]	[4.1792]
Size	0.160^***^	0.140^***^	0.136^***^	0.182^***^
	[0.0096]	[0.0095]	[0.0093]	[0.0141]
Lev	−0.086^**^	−0.123^***^	−0.108^***^	−0.0822^*^
	[0.0383]	[0.0376]	[0.0368]	[0.0480]
ROE	−0.338^***^	−0.340^***^	−0.338^***^	−0.362^***^
	[0.0751]	[0.0742]	[0.0740]	[0.0786]
Cash	0.224^***^	0.186^***^	0.180^***^	0.173^***^
	[0.0306]	[0.0306]	[0.0304]	[0.0329]
Lgcom	0.188^***^	0.187^***^	0.177^***^	0.168^***^
	[0.0103]	[0.0100]	[0.0099]	[0.0125]
Shr	−0.164^***^	−0.126^***^	−0.136^***^	−0.270^***^
	[0.0491]	[0.0466]	[0.0451]	[0.0802]
CCons	−0.063^***^	−0.0676^***^	−0.0754^***^	−0.0611^***^
	[0.0045]	[0.0044]	[0.0044]	[0.0047]
Vepop	−0.000^***^	−0.000^***^	−0.000^***^	−0.000^***^
	[0.0000]	[0.0000]	[0.0000]	[0.0000]
Buiope	−0.018^***^	−0.020^***^	−0.018^***^	−0.019^***^
	[0.0016]	[0.0015]	[0.0015]	[0.0019]
Cons	11.980^***^	13.710^***^	14.470^***^	11.760^***^
	[0.5205]	[0.5147]	[0.5203]	[0.6287]
Soe	0.154^***^	0.145^***^	0.141^***^	Yes
	[0.0179]	[0.0169]	[0.0161]	
Industry	No	Yes	Yes	Yes
Province	No	No	Yes	Yes
Enterprise	No	No	No	Yes
Adjusted *R*^2^	0.229	0.227	0.227	0.232
Chi-square	2099.10^***^	10453.49^***^	13042.66^***^	
*F*-value				146.56^***^

*N = 21,372*,

* *p < 0.01*,

** *p < 0.05*,

*** *p < 0.1, and the standard deviation is shown in square brackets*.

It can be seen from Table 5 (1) that after controlling the relevant factors affecting air quality, environmental regulation and air quality are significantly positively correlated at 1% level, which means that environmental regulation first increases the pollution cost and environmental technology. The local air quality has been greatly improved. In columns (2)–(4), the industry province and firm fixed effects are further controlled, and environmental regulation and air quality are still significantly positively correlated at the 1% level. This explains that under other conditions, areas with higher environmental regulation intensity have better local air quality. H2 is proven.

**Table 5:** Relationship between environmental regulation and air quality.

**Variables**	**Random effect**			**Fixed effect**
	**Air (1)**	**Air (2)**	**Air (3)**	**Air (4)**
Hr	7.718^***^	8.166^***^	38.880^***^	38.790^***^
	[1.0393]	[1.0537]	[1.9312]	[1.9830]
InGDP	−0.129^***^	−0.123^***^	−0.106^***^	−0.0814^***^
	[0.0135]	[0.0137]	[0.0208]	[0.0222]
CCons	−0.080^***^	−0.080^***^	−0.086^***^	−0.089^***^
	[0.0015]	[0.0015]	[0.0015]	[0.0015]
Vepop	−0.000	−0.000	−0.000	−0.000^***^
	[0.0000]	[0.0000]	[0.0000]	[0.0000]
Buiope	0.001^***^	0.001^***^	0.000	0.001
	[0.0003]	[0.0003]	[0.0002]	[0.0006]
Cons	8.391^***^	8.364^***^	8.804^***^	9.284^***^
	[0.1482]	[0.1490]	[0.1499]	[0.1529]
Industry	No	Yes	Yes	Yes
Province	No	No	Yes	Yes
Enterprise	No	No	No	Yes
Adjusted *R*^2^	0.134	0.134	0.152	0.152
Chi-square	3659.25^***^	7230.91^***^	54573.04^***^	
*F*-value				789.26^***^

*N = 21,372*,

*** *p < 0.1, and the standard deviation is shown in square brackets*.

Table 6 (1) shows that after controlling the relevant factors affecting employee compensation, air quality, and employee compensation are significantly negatively correlated at the 1% level. In columns (2)–(4), further control industry fixed effects, provincial fixed effects, corporate fixed effects, environmental regulation, and air quality are still significantly negatively correlated at the 1% level. This explains that under other conditions, when the air quality is improved, the salary level of local enterprises will be relatively low, and H3 is proven. This means that the better the air quality, the higher the non-monetary income, which reduces the risk premium (currency pay) compensation required by the employees due to high environmental risks. Therefore, in areas with better air quality, the monetary compensation level of employees is lower. In summary, environmental regulations have improved air quality, which in turn has reduced employees' premium compensation for environmental risks; that is, the reduction of employee compensation.

**Table 6:** Relationship between air quality and employee compensation.

**Variables**	**Random effect**			**Fixed effect**
	**Lgwage (1)**	**Lgwage (2)**	**Lgwage (3)**	**Lgwage (4)**
Air	−0.045^***^	−0.047^***^	−0.067^***^	−0.069^***^
	[0.0128]	[0.0127]	[0.0128]	[0.0129]
Size	0.160^***^	0.139^***^	0.136^***^	0.182^***^
	[0.0096]	[0.0095]	[0.0093]	[0.0141]
Lev	−0.0851^**^	−0.124^***^	−0.106^**^	−0.0798^*^
	[0.0384]	[0.0376]	[0.0368]	[0.0480]
ROE	−0.325^***^	−0.328^***^	−0.324^***^	−0.348^***^
	[0.0751]	[0.0742]	[0.0739]	[0.0785]
Cash	0.228^***^	0.188^***^	0.181^***^	0.175^***^
	[0.0305]	[0.0305]	[0.0303]	[0.0327]
Lgcom	0.190^***^	0.188^***^	0.178^***^	0.168^***^
	[0.0103]	[0.0100]	[0.0099]	[0.0125]
Shr	−0.166^***^	−0.126^***^	−0.139^***^	−0.276^***^
	[0.0490]	[0.0465]	[0.0451]	[0.0802]
CCons	−0.066^***^	−0.071^***^	−0.081^***^	−0.066^***^
	[0.0047]	[0.0047]	[0.0047]	[0.0050]
Vepop	−0.000^***^	−0.000^***^	−0.000^***^	−0.000^***^
	[0.0000]	[0.0000]	[0.0000]	[0.0000]
Buiope	−0.018^***^	−0.020^***^	−0.019^***^	−0.019^***^
	[0.0016]	[0.0015]	[0.0015]	[0.0019]
Cons	12.250^***^	14.030^***^	14.940^***^	12.290^***^
	[0.5467]	[0.5406]	[0.5484]	[0.6557]
Soe	0.151^***^	0.143^***^	0.141^***^	Yes
	[0.0180]	[0.0169]	[0.0162]	
Industry	No	Yes	Yes	Yes
Province	No	No	Yes	Yes
Enterprise	No	No	No	Yes
Adjusted *R*^2^	0.229	0.227	0.227	0.232
Chi-square	2068.96^***^	10396.05^***^	13036.55^***^	
*F*-value				146.04^***^

*N = 21,372*,

* *p < 0.01*,

** *p < 0.05*,

*** *p < 0.1, and the standard deviation is shown in square brackets*.

Tables 7, 8 indicate that after controlling the relevant factors affecting the financial performance of the enterprise, as well as the fixed effects of the industry, province, and the enterprise, employee compensation, and the financial performance of the enterprise are significantly negatively correlated at the 1% level. It can be seen from Table 9 that after controlling the relevant factors affecting the growth of the enterprise, as well as the fixed effects of the industry, province, and the enterprise, the employee compensation, and the enterprise growth index are significantly negatively correlated at the 1% level. In summary, it shows that the low salary level brought by environmental regulation is conducive to the improvement of corporate performance. H4 is proven.

**Table 7:** Relationship between employee compensation and return on net assets.

**Variables**	**Random effect**			**Fixed effect**
	**ROE (1)**	**ROE (2)**	**ROE (3)**	**ROE (4)**
Lgwage	−0.006^***^	−0.007^***^	−0.007^***^	−0.013^***^
	[0.0016]	[0.0017]	[0.0018]	[0.0028]
Size	0.005^***^	0.004^***^	0.004^***^	0.009^***^
	[0.0009]	[0.0009]	[0.0009]	[0.0019]
Lev	−0.144^***^	−0.147^***^	−0.146^***^	−0.179^***^
	[0.0048]	[0.0050]	[0.0050]	[0.0081]
Cash	−0.024^***^	−0.025^***^	−0.024^***^	−0.040^***^
	[0.0054]	[0.0055]	[0.0056]	[0.0070]
Lgcom	0.013^***^	0.013^***^	0.013^***^	0.005^**^
	[0.0011]	[0.0011]	[0.0011]	[0.0019]
Shr	0.070^***^	0.070^***^	0.068^***^	0.097^***^
	[0.0045]	[0.0046]	[0.0046]	[0.0115]
CCons	0.002^**^	0.001	0.002^**^	0.002^***^
	[0.0007]	[0.0007]	[0.0007]	[0.0008]
Vepop	−0.000	−0.000	−0.000	−0.000
	[0.0000]	[0.0000]	[0.0000]	[0.0000]
Buiope	0.000^**^	0.000^**^	0.000^**^	0.001^***^
	[0.0002]	[0.0002]	[0.0002]	[0.0003]
Cons	−0.314^***^	−0.238^***^	−0.246^***^	−0.273^***^
	[0.0776]	[0.0800]	[0.0833]	[0.1059]
Soe	−0.009^***^	−0.008^***^	−0.007^***^	Yes
	[0.0014]	[0.0014]	[0.0015]	
Industry	No	Yes	Yes	Yes
Province	No	No	Yes	Yes
Enterprise	No	No	No	Yes
Adjusted *R*^2^	0.120	0.121	0.121	0.127
Chi-square	1852.74^***^	17306.27^***^	18218.77^***^	
*F*-value				63.03^***^

*N = 21,372*,

** *p < 0.05*,

*** *p < 0.1, and the standard deviation is shown in square brackets*.

**Table 8:** Relationship between employee compensation and total return on assets.

**Variables**	**Random effect**			**Fixed effect**
	**ROA (1)**	**ROA (2)**	**ROA (3)**	**ROA (4)**
Lgwage	−0.006^***^	−0.007^***^	−0.008^***^	−0.012^***^
	[0.0018]	[0.0019]	[0.0020]	[0.0029]
Size	0.004^***^	0.004^***^	0.004^***^	0.008^***^
	[0.0010]	[0.0010]	[0.0010]	[0.0020]
Lev	−0.154^***^	−0.157^***^	−0.156^***^	−0.181^***^
	[0.0050]	[0.0051]	[0.0051]	[0.0081]
Cash	−0.046^***^	−0.047^***^	−0.046^***^	−0.066^***^
	[0.0054]	[0.0055]	[0.0055]	[0.0068]
Lgcom	0.014^***^	0.013^***^	0.013^***^	0.005^*^
	[0.0012]	[0.0012]	[0.0012]	[0.0019]
Shr	0.080^***^	0.078^***^	0.077^***^	0.104^***^
	[0.0051]	[0.0051]	[0.0051]	[0.0120]
CCons	0.002^*^	0.002^*^	0.002^*^	0.002^*^
	[0.0007]	[0.0007]	[0.0008]	[0.0008]
Vepop	−0.000	0.000	0.000	−0.000
	[0.0000]	[0.0000]	[0.0000]	[0.0000]
Buiope	0.000^*^	0.000	0.000^*^	0.001^**^
	[0.0002]	[0.0002]	[0.0002]	[0.0003]
Cons	−0.309^***^	−0.233^**^	−0.230^**^	−0.235^*^
	[0.0801]	[0.0823]	[0.0853]	[0.1061]
Soe	−0.012^***^	−0.011^***^	−0.010^***^	Yes
	[0.0015]	[0.0016]	[0.0016]	
Industry	No	Yes	Yes	Yes
Province	No	No	Yes	Yes
Enterprise	No	No	No	Yes
Adjusted *R*^2^	0.127	0.128	0.128	0.135
Chi-square	1844.66^***^	15225.43^***^	16058.56^***^	
*F*-value				66.13^***^

*N = 21,372*,

* *p < 0.01*,

** *p < 0.05*,

*** *p < 0.1, and the standard deviation is shown in square brackets*.

**Table 9:** Relationship between employee compensation and operating income growth rate (OIGR).

**Variables**	**Random effect**			**Fixed effect**
	**OIGR (2)**	**OIGR (2)**	**OIGR (3)**	**OIGR (4)**
Lgwage	−0.020^**^	−0.033^***^	−0.037^***^	−0.140^***^
	[0.0081]	[0.0087]	[0.0091]	[0.0192]
Size	0.026^***^	0.029^***^	0.031^***^	0.143^***^
	[0.0043]	[0.0045]	[0.0046]	[0.0137]
Lev	−0.049^**^	−0.049^**^	−0.045^**^	−0.039
	[0.0214]	[0.0220]	[0.0224]	[0.0515]
Cash	−0.266^***^	−0.296^***^	−0.302^***^	−0.321^***^
	[0.0259]	[0.0266]	[0.0270]	[0.0436]
Lgcom	0.033^***^	0.029^***^	0.024^***^	0.022
	[0.0055]	[0.0056]	[0.0057]	[0.0131]
Shr	−0.079^***^	−0.067^***^	−0.070^***^	−0.001
	[0.0195]	[0.0196]	[0.0196]	[0.0647]
CCons	−0.033^***^	−0.035^***^	−0.040^***^	−0.023^***^
	[0.0053]	[0.0052]	[0.0055]	[0.0059]
Vepop	−0.000^***^	−0.000^***^	−0.000^***^	−0.000^***^
	[0.0000]	[0.0000]	[0.0000]	[0.0000]
Buiope	−0.005^***^	−0.005^***^	−0.004^***^	0.002
	[0.0009]	[0.0009]	[0.0009]	[0.0020]
Cons	2.874^***^	3.070^***^	3.583^***^	0.629
	[0.5504]	[0.5539]	[0.5820]	[0.7142]
Soe	−0.086^***^	−0.079^***^	−0.079^***^	Yes
	[0.0068]	[0.0071]	[0.0073]	
Industry	No	Yes	Yes	Yes
Province	No	No	Yes	Yes
Enterprise	No	No	No	Yes
Adjusted *R*^2^	0.0289	0.0313	0.0315	0.0478
Chi-square	550.30^***^	4134.53^***^	4288.10^***^	
*F*-value				34.89^***^

*N = 21,372*,

** *p < 0.05*,

*** *p < 0.1, and the standard deviation is shown in square brackets*.

## Conclusions and Discussion

The previous literature on environmental regulation mainly focuses on the impact of relevant measures on the technological innovation and investment behavior of enterprises (4–8, 10). This study takes the employee compensation as the starting point and empirically analyzes the effect of environmental improvement brought by environmental regulation on the non-monetary benefits of employees, which will help identify and evaluate the impact of environmental regulation on enterprise development.

The main findings of this study are that environmental regulation can improve air quality, and air quality affects the employee's salary, which in turn affects regional economic development. Air quality is one of the important factors affecting human society. Good air will enhance the employees' physical and mental health, improve their work efficiency, motivate employee to work hard, and reduce the labor cost of the enterprise. It can be seen that the pursuit of a good ecological environment and efficient economic development can be a win–win situation.

Environmental regulation can improve the ecological environment and corporate performance. The findings of this research have important implications for the healthy development of society. The results of environmental regulations that have improved the ecological environment show that environmental regulation can effectively meet people's health and safety needs. It is found that the role of environmental regulation in improving corporate performance is also of great significance to communities and residents. Enterprises are an important part of the community. A good level of performance enables companies to better fulfill their responsibilities to the community and enhance the residents' welfare, such as by donating funds to build community public facilities, improving community education, and protecting the employment of community residents. Regulations can provide the above benefits.

Therefore, the findings of this study have important policy implications. Local governments should strive to implement the concept of “lucid waters and lush mountains are invaluable assets” and formulate environmental regulation policies in line with local conditions. Simultaneously, the policy will be implemented, the pollution control system will be continuously improved, the pressure on the environment caused by pollution will be alleviated, and higher quality public goods and services will be provided. At the same time, enterprises should pay attention not only to the development of economic benefits but also to environmental protection, active improvement of production methods, reduction in pollution emissions through process innovation, and formation of an efficient growth model with harmonious and friendly enterprises at the core. Enterprises should also actively protect the health rights of employees and improve their working environment, so as to encourage employees to work hard; otherwise, the deterioration of the working environment will affect the health and enthusiasm of employees.

This study only focuses on the income of ordinary employees. Relatively speaking, their income is low, but employees are also a vulnerable group in the enterprise. It is difficult for ordinary workers to migrate due to air quality problems, so they have strong incentives for companies to pay money to compensate for non-monetary gains. However, in the same situation, highly skilled employees and senior executives have a strong ability to evade air pollution, that is, the possibility of fleeing from heavily polluted areas is high. The difference in regional air quality will lead to the reallocation of human resources among regions, which will cause the loss of human resources in enterprises with severe pollution. How does the regulation of environmental pollution affect the flow of high-end human resources, and how does it affect corporate performance? This issue was not included in the research.

Pollution control is a relatively long process, and it is still difficult to retain highly skilled employees in heavily polluted cities during periods of environmental improvement. During periods of environmental improvement, what is the extent of human resources loss of enterprises in polluted areas? How can government and enterprises alleviate the human resource loss effect of enterprises in heavily polluted areas and continuously improve corporate performance? These are important topics that merit additional research in the future.

## Data Availability Statement

The datasets generated for this study are available on request to the corresponding author.

## Author Contributions

K-CChai: determine the title of the article and the main research methods, and propose amendments to the article. YH: writing of the article, data search, data processing, using the model for research and analysis, and making recommendations for the analysis results. K-CChan: guide the ideas of this paper. All authors designed and conducted the study, analyzed the data, drafted the manuscript, contributed to the interpretation of results, critically reviewed the draft, and approved the final manuscript.

### Conflict of Interest

The authors declare that the research was conducted in the absence of any commercial or financial relationships that could be construed as a potential conflict of interest.
